# Primary Hepatocellular Carcinoma: Hospital Based Epidemiologic Study

**DOI:** 10.1155/1990/84213

**Published:** 1990

**Authors:** Jeffrey D. Sedlack, Russell J. Nauta

**Affiliations:** 1 Department of Surgery Georgetown University School of Medicine USA; 2 M.D. 3800 Reservoir Rd NW 4th Floor PHC Bldg Washington DC 20007 USA

## Abstract

From 1968-1985 a series of thirty-seven patients with primary hepatocellular carcinoma was collected from the tumor registry of the Fairfax County Hospital, in the metropolitan Washington, D.C. area. These patients were found to have a mean age at diagnosis of sixty-two (males) to sixty-six (females). Thirty per cent of patients were previously cirrhotic and nineteen per cent had a history of viral hepatitis. There were no patients with documented birth control pill or steroid use. The most common presenting symptoms were anorexia and right upper quadrant pain. Liver-spleen scan was the most commonly used diagnostic study, but by the 1980's CT scanning was usually diagnostic. Both alkaline phosphatase and serum glutamyloxalotransferase were reliably elevated in twenty-six of twenty-eight and twenty-one of twenty-four patients respectively. Forty-eight per cent of patients with tumor histology reprted had multicentric tumors, thirty-eight per cent had nodular tumors, and fourteen per cent had diffuse disease.

Survival was as dismal in this as in other studies with a mean of seventy-nine days. No significant difference was noted between cirrhotic and non-cirrhotic patients. Chemotherapy and radiation therapy did not significantly impact upon survival.

Finally, a cohort analysis was done and a possibly significant peak in incidence of primary hepatocellular carcinoma was seen in men born from about 1911 through 1920. The authors noted that these males were in the group of draft eligible persons for World War II and questioned a link between veteran status and later development of HCC.


*HPB Surgery*, 1990, Vol. 3, pp. 11–19
Reprints available directly from the publisher
Photocopying permitted by license only

© 1990 Harwood Academic Publishers GmbH
Printed in the United Kingdom

### PRIMARY HEPATOCELLULAR CARCINOMA: Hospital Based Epidemiologic Study

JEFFREY D. SEDLACK and RUSSELL J. NAUTA*
*Department of Surgery Georgetown University School of Medicine*

*(Received 10 January 1990)*

From 1968–1985 a series of thirty-seven patients with primary hepatocellular carcinoma was collected from the tumor registry of the Fairfax County Hospital, in the metropolitan Washington, D.C. area. These patients were found to have a mean age at diagnosis of sixty-two (males) to sixty-six (females). Thirty per cent of patients were previously cirrhotic and nineteen per cent had a history of viral hepatitis. There were no patients with documented birth control pill or steroid use. The most common presenting symptoms were anorexia and right upper quadrant pain. Liver-spleen scan was the most commonly used diagnostic study, but by the 1980's CT scanning was usually diagnostic. Both alkaline phosphatase and serum glutamyloxalotransferase were reliably elevated in twenty-six of twenty-eight and twenty-one of twenty-four patients respectively. Forty-eight per cent of patients with tumor histology reprted had multicentric tumors, thirty-eight per cent had nodular tumors, and fourteen per cent had diffuse disease.

Survival was as dismal in this as in other studies with a mean of seventy-nine days. No significant difference was noted between cirrhotic and non-cirrhotic patients. Chemotherapy and radiation therapy did not significantly impact upon survival.

Finally, a cohort analysis was done and a possibly significant peak in incidence of primary hepatocellular carcinoma was seen in men born from about 1911 through 1920. The authors noted that these males were in the group of draft eligible persons for World War II and questioned a link between veteran status and later development of HCC.

KEY WORDS: Carcinoma, liver, incidence, carcino-embryonic antigen, alpha-fetoprotein, veteran, hepatitis, cirrhosis

### SHORT SUMMARY

Primary hepatocellular carcinoma was examined in a hospital based epidemiologic study. Demographic statistics were found to correlate with North American averages. Two survivors were identified, both of whom underwent resection. A cohort analysis was done which showed a peak in incidence of primary liver cancer in males born between 1910–1920. A possible link with World War II veteran status was postulated.

### INTRODUCTION

Primary hepatocellular carcinoma is the most common malignancy worldwide with a very high incidence in Africa and the Far East.[1,8] Various environmental, toxic,

*All correspondence should be addressed to: Russell J. Nauta, M.D. 3800 Reservoir Rd NW, 4th Floor PHC Bldg, Washington, DC 20007
Study done at the Fairfax County Hospital, Fairfax, Virgina and infectious agents have been statistically and experimentally shown to be relevant in the genesis of the disease. Chief among these are hepatitis B virus[5,6,8,16] and alcoholic cirrhosis.[1,8,10,12,16,17] Because of the low incidence of this disease, however, and because of its uniformly rapid fatality, few North American series have been published. This report is a study of a series of patients collected at the Fairfax County Hospital in the Washington, D.C., metropolitan area.

### MATERIALS AND METHODS

Records of the tumor registry of the Fairfax County Hospital, a five hundred bed private hospital in suburban Washington, D.C., were searched for both clinical and autopsy diagnoses of primary hepatocellular carcinoma. Such records have been kept at the hospital since 1968, thus a time frame of 1968–1985 was used for this study. Following patient identification, hospital records were abstracted for demographic, pathologic, etiologic and diagnostic information. This data was then collated to produce this report.

### RESULTS

A total of 38 patients with a diagnosis of primary hepatocellular carcinoma were identified. After examination of pathology reports, hospital records, and office charts, one patient was eliminated from the study with a diagnosis of metastatic colon cancer leaving a total of thirty-seven patients for analysis.

Of the thirty-seven patients in the study there were twenty-three males and fourteen females (M:F ratio 1.6:1). Thirty-four patients were Caucasian, one black and two Oriental. The two Oriental patients were both immigrants from Korea in adulthood; one Caucasian was originally from the Dominican Republic. The remaining thirty-four patients were native. The mean age of onset was in the sixth decade in males (mean = 62), and slightly older for females (mean = 66). A limited cohort analysis was done comparing native patients with the diagnosis of liver cancer by sex and by birth year. A peak in incidence in males born 1911–1925 was shown (Figure 1). Tumor pattern was reported in twenty-nine of thirty-seven patients. Fourteen (48%) had multicentric tumors, eleven (38%) had nodular tumors, and four (14%) had diffuse tumor.

The series was examined for co-morbid disease. (Table 1) Eleven patients were reported as cirrhotic (30%). Of these, five had Laennec's cirrhosis, two had post-hepatitic cirrhosis, and four patients had cirrhosis without pathologic type reported. Seven patients were described as alcoholic (19%). Of these seven patients, four had both documented alcoholism and cirrhosis. Seven patients had a history of hepatitis of which six had hepatitis B, and two had known chronic active hepatitis. No patients had documented birth control pill, or steroid use, and there were no patients with hemochromatosis. One of the Korean patients had viral cirrhosis, the other had alcoholic liver disease. The patient from the Dominican Republic had alpha-1-antitrypsin deficiency. Two patients (5%) had a history of cholelithiasis, and eight patients had other malignancies of widely varying and non-duplicated types.

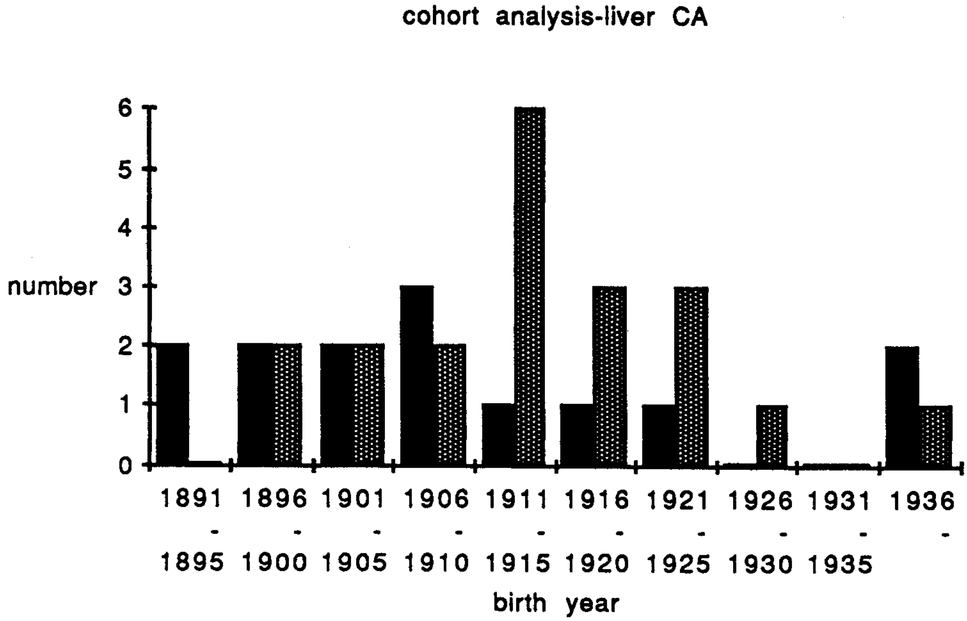

**Figure 1** Cohort analysis (birth year group vs. number in group).

**Table 1** Other Medical Problems.

| | | | |
|---|---|---|---|
| Cirrhosis | | 11 (30%) | |
| | Laennec's | | 5 |
| | Post Hepatitic | | 2 |
| | Unknown Pathology | | 4 |
| Alcoholism | | 7 (10%) | |
| | alcoholism with cirrhosis documented | | 4 |
| Viral Hepatitis | | 7 (19%) | |
| | Chronic Active Hepatitis | | 2 |
| Other Malignancies | | 8 (23%) | |
| Cholelithiasis | | 2 (5%) | |
| Hemochromatosis | | 0 (0%) | |
| BCP/steroid use documented | | 0 (0%) | |

Evaluation of presenting symptoms (Table 2) from these patients showed that the most common symptoms, were anorexia and abdominal pain, usually originating in the right upper quadrant. Nearly one-quarter of the patients had an abdominal mass at presentation. Two patients entered with a rapid decompensation of previously stable cirrhosis, a symptom suggested by many authors to be a hallmark of liver cancer in the cirrhotic. One patient, admitted with advanced disease complained principally of rib pain from tumor erosion.

Many diagnostic studies are open to the clinician in the diagnosis of hepatocellular carcinoma (Table 3). In this study the most frequently utilized was nuclear liver scanning reflecting the available technology of the late 1960's and early 1970's. By the late 1970's CT scanning was very frequently used and there was a decline in the frequency of liver scanning. Three patients in our series were diagnosed only at autopsy, a rate comparable to other American studies.[6] Peritoneioscopy with liver biopsy under direct visualization was done in only two patients, but with pathologic diagnoses made in both.

There was a low rate of reporting of both Carcino-Embryonic Antigen (CEA) and Alpha-fetoprotein (AFP) levels in our series (Table 3). One half of the AFP levels reported were said to be normal. CEA levels were somewhat more reliably elevated. SGOT and Alkaline Phosphatase levels were also found to be significantly elevated in the majority of patients.

**Table 2** Presenting Symptoms.

| | |
|---|---|
| Anorexia | 25 (68%) |
| Abdominal Pain | 21 (57%) |
| Palpable Mass | 9 (24%) |
| Ascites | 7 (19%) |
| Hemorrhage | 3 (8%) |
| Jaundice | 3 (8%) |
| Decompensating Cirrhosis | 2 (5%) |
| Rib Pain | 1 (3%) |

**Table 3** Diagnostic Studies.

| | |
|---|---|
| liver scan | 15 (41%) |
| percutaneous biopsy | 14 (38%) |
| laparotomy | 12 (32%) |
| peritoneoscopy | 2 (5%) |
| ultrasound | 1 (3%) |
| diagnosis at autopsy | 3 (8%) |
| alpha-fetoprotein<br>reported in 14 patients | 7 negative (or less than 5)<br>median value 100 |
| Serum Glutamyloxalo-<br>transferase (SGOT)<br>reported in 24 patients<br>elevated in 21 patients | normal 10–41<br>range 18–526<br>average 125 |
| Alkaline Phosphatase<br>reported in 28 patients<br>elevated in 26 patients | normal 30–110<br>range 85–1585<br>average 465 |
| Carcinoembryonic antigen<br>reported in 13 patients | negative in 1<br>range 0–23.5<br>average 6.1 |

Approximately one-half of patients in the series underwent some form of treatment. Three patients underwent liver resection, with one documented cure in a young female patient undergoing liver resection in the early 1970's. Another patient, an elderly woman, underwent pre-operative chemotherapy and, several years later resection of liver tumor mass surrounded by tumor free necrotic margins. The third operative patient died perioperatively. Chemotherapy and radiation therapy were used in a number of patients, usually as a final effort and with no prolongation of life.

An analysis of survival was done (Table 4). The two cures were excluded from survival data as were the three patients diagnosed at autopsy. The remaining patients were also further divided into cirrhotic and non-cirrhotic groups because of a slightly shorter reported survival in cirrhotic patients than in non-cirrhotic patients. For the entire group, the survival range was one, to three-hundred and fifteen days with an average of seventy-nine days. Non-cirrhotic patients tended to live somewhat longer than the mean at ninety-three days and cirrhotic patients somewhat less at forty-two days, though this was not statistically signficant by $X^2$ analysis of variance.

### DISCUSSION

Patients with primary hepatocellular carcinoma in North America present a difficult epidemiologic problem given their small incidence and quick demise. This investigation of liver cancer in a suburban/metropolitan hospital in Virginia, nonetheless yielded data for comparison with other North American studies and provided a useful contrast with international data (Table 5). A cohort analysis was done, which we have not seen elsewhere. Our study, though constrained by the age skew of the hospitalized population from 1968–1985, showed a seemingly significant peak in occurence of primary hepatocellular carcinoma in the group of native males born from 1911 through 1925. Over one-half of our patients came from this

**Table 4** Survival (excluding 2 "cures" and 3 diagnosed at autopsy = 32 patients).

| | |
|---|---|
| *Overall* | |
| Range | 1–315 Days |
| Average | 78.8 Days |
| Standard Deviation | 74.12 Days |
| *Non-Cirrhotic Patients* (N = 23) | |
| Range | 1–315 Days |
| Average | 93.17 Days |
| Standard Deviation | 80.8 Days |
| *Cirrhotic Patients* (N = 9) | |
| Range | 1–89 Days |
| Average | 42 Days |
| Standard Deviation | 35 Days |

**Table 5** Institutional Experiences with Hepatocellular Carcinoma.

| *Study* | *# Patients*<br>*Avg.Age*<br>*W o B*<br>*M/F* | *Cirrhosis* | *Etoh* | *Viral Hep.*<br>*CAH* | *Other Malig.* | *AFP* |
|---|---|---|---|---|---|---|
| Luna<br>Seattle | 35<br>N.A.<br>28/5/2<br>31/4 | 27 (77%) | 16 (46%) | 5<br>4 | 8 (23%) | N.A. |
| Naga-<br>Sue<br>Japan | 100<br>N.A. | 78 (78%) | 39 (39%) | 43 (43%)<br>24 | N.A. | +54/83 |
| L.C.S.G.<br>Japan | 1047<br>55.9<br>0/1047/0<br>858/189 | 395 (44.8) | 158 (15%) | 457 (55%)<br>269 | N.A. | N.A. |
| Lai<br>Hong<br>Kong | 211<br>"60'S"<br>0/211/0<br>177/34 | 12 (6%) | 23 (11%) | N.A.<br>19/20 | N.A. | N.A. |
| Inouye<br>Hi, USA | 205<br>61<br>58/136/0<br>161/44 | 111 | N.A. | N.A.<br>10/10 | | +9/30 |
| Chlebo-<br>Wski<br>L.A., USA | 121<br>52<br>722/26/30<br>90/31 | (63%) | (31–55%) | (52%) | N.A. | N.A. |
| Sedlack<br>D.C., USA | 37<br>64<br>34/2/1<br>23/14 | 11 (30%) | 7 (19%) | 7 (19%)<br>2 | 8 (22%) | +7/14 |

group, a subset of males whose life was likely to have included service in World War II. Seeff, *et al.*[19] have reported on the development of viral hepatitis in American veterans of that war who received the Yellow Fever vaccination. As "yellow jaundice" was endemic in troops stationed in Asia during that time, further studies are indicated to determine if the veterans of the Pacific War are at an increased risk of late development of hepatocellular carcinoma.

This study again pointed out the importance of liver disease, principally alcoholic cirrhosis and viral hepatitis, in the genesis of primary liver carcinoma. Our percentage of cirrhosis is lower than in other North American studies. There are equal numbers of patients with alcoholic and with viral liver disease in our group, comparable to other North American studies.[1,6]

Only two patients were seropositive for hepatitis B surface antigen as compared with higher rates in both Seattle[6] and Los Angeles.[1] Given the high rate of finding hepatitis B virus genome incorporated into hepatocellular carcinoma and other "normal" liver tissue in patients with the cancer,[14] this raises the obvious question of whether many tumor promoters exist for primary liver cancer.

No relationship was found between blood group and risk for primary liver carcinoma, distinguishing liver carcinoma from many of the gastrointestinal tumors with their predilection for a certain blood group, particularly group A.

Several studies have shown a significant number of patients with hepatocellular carcinoma to have a history of other malignancies.[6] This was demonstrated again in this study, but the malignancies were all differing types and none contributed to the patients's eventual demise. We feel that given the high frequency of all types of malignancy in the United States, the age of the population at risk for hepatocellular cancer, and the scattershot distribution of other malignancies seen here, no satisfactory link may be discerned.

The use of Alpha-fetoprotein measurements as a diagnostic tool cannot be assessed from this study. Only fourteen out of thirty-five patients had the test, reflecting the time frame of the study. The rate of positive testers of fifty per cent compares unfavorably to other published reports from both the Orient and the United States of seventy to one-hundred per cent positive[18] though it is known to be most useful in screening the population with a viral carcinogen. Survival data in those patients show an overall mean survival of under three months, with no statistically demonstrable survival difference between cirrhotic and non-cirrhotic patients, in contrast with several studies which show a slight decrease in survival of cirrhotic patients.[1]

Many issues are relevant to the study of hepatocellular carcinoma. Our study showed an increased risk of carcinoma in males born between 1911 and 1925 for unknown but possibly statistical reasons, and the standard North American equal ratio of alcoholic to viral liver disease as predisposing conditions. More study of the risks of hepatocellular carcinoma in North American males is needed to determine their risk factors beyond ethanol and hepatitis especially with regard to veteran status and travel history.

### *References*

1. Chlebowski, R., Tong, M., Weissman, J., *et al.*, (1984) Hepatocellular Carcinoma: diagnostic and prognostic features in North American patients. *Cancer*, **53**
2. Inouye, A. and Whelan, T. (1979) Primary liver cancer: A review of 205 cases in Hawaii. *Amer. Jrnl. Surg.*, **138**, 53–61
3. King, H. and Locke, F.B. (1980) Cancer mortality among Chinese in the United States. *Jrnl. Natl. Cancer, Inst.*, **65**, 1141–1148
4. Lai, C., Lam, K., Wong, K., Wu, P. and Todd, D. (1981) Clinical features of hepatocellular carcinoma: review of 211 patients in Hong Kong. *Cancer*, **47**, 2746–2755
5. The Liver Cancer Study Group of Japan (1984) Primary liver cancer in Japan. *Cancer*, **54**, 1747–1755
6. Luna, G., Florence, L., Joyhnasen, K. (1985) Hepatocellular carcinoma: A 5 year institutional experience. *Amer. Jrnl. Surg.* **149**, 591–594
7. Lee, Y. (1983) Primary carcinoma of the liver: Diagnosis, prognosis and management (1983). *Jrnl. Sur. Oncol.*, **22**, 17–25
8. Cook-Mozaffari, P. and Van Rensburg, S. (1984) Cancer of the Liver. *Brit. Med. Bult.*, **40**, 342–345
9. Palmer-Beasley, H., Hwanag, L., Lin, C., Chien, C. (1981) Hepatocellular carcinoma and hepatitis B virus. *Lancet*, **2**, 1129–1133
10. Brechot, C., Nalpas, B., Couroce, A., *et al.*, (1982) Evidence that hepatitis B virus has a role in liver-cell carcinoma in alcoholic liver disease. *New Eng. Jrnl. Med.*, **306**, 1384–1387
11. Gilliam, J., Geisinger, K. and Richter J. (1984) Primary hepatocellular carcinoma after chronic non-A, non-B post-transfusion hepatitis. *Annls. Intnl. Med.*, **101**, 794–795
12. Johnson, P., Krasner, N., Portmann, B., Eddleston, A. and Williams, R. (1978) Hepatocellular carcinoma in Great Britain: influence of age, sex HBsAG status and etiology of underlying cirrhosis. *Gut*, **19**, 1022–1026

13. Omata, M., Ashcavai, M., Liew, C. and Peters, R. (1979) Hepatocellular carcinoma in the U.S.A., etiologic considerations. *Gastro*, **76**, 279–287
14. Shafritz, D., Shouval, D., Sherman, H., Hadziyannis, S., and Kew, M. (1981) Integration of Hepatitis B virus DNA into the Genome of liver cells in chronic liver disease and hepatocellular carcinoma. *New Eng. Jrnl. Med.*, **305**, 1067–1073
15. Tiollais, P., Charnay, P. and Vyas, G. (1981) Biology of hepatitis B virus. *Science*, **213**, 406–411
16. Zaman, S., Melia, W., Johnson, R., Portmann, B., Johnson, P. and Williams, R. (1985) Risk factors in development of hepatocellular carcinoma in cirrhosis: prospective study of 613 patients. *Lancet*, **1**, 1357–1359
17. Nagasue, N., Yukaya, H., Hamada, T., Hirose, S., Kanashima, R., Inokuchi, K. (1984) The natural history of hepatocellular carcinoma: a study of 100 untreated cases. *Cancer*, **54**, 1461–1465
18. Wepsic, H. and Kirkpatrick, A. (1984) Alpha-fetoprotein and its relevance to human disease. *Gastro*, **77**, 787–796
19. Seeff, L., *et al.* (1987) A serologic follow-up of the 1942 epidemic of post-vaccination hepatitis in the United States Army. *New Eng. Jrnl. Med.*, **316**, 965–970

(*Accepted by S. Bengmark on 8 January 1990*)

### INVITED COMMENTARY

It is very interesting that this paper concerns the epidemiologic and clinicopathological studies of hepatocellular carcinoma in one of the areas with small incidence of this disease.

In Japan, hepatocellular carcinoma is one of the most fatal malignant diseases. According to the collected series by the Liver Cancer Study Group of Japan, about 80% of the patients had liver cirrhosis, especially post-hepatitis type, among 2300 cases of hepatocellular carcinoma diagnosed in Japan during 2 years from Jan. 1, 1984 to Dec. 31, 1985. Of the patients with liver cirrhosis, hepatitis B surface antigen was positive in only 24.6%.

In the suburban hospital, Washington D.C., shown in this paper, only 30% of the patients had liver cirrhosis. This figure is much smaller than in Japan. Because hepatocellular carcinoma in this series were considered to be far advanced and large in size, liver-spleen scan was very valuable for diagnosis. However, liver-spleen scan is certainly not reliable for diagnosis of a minute hepatocellular carcinoma in Japan. Ultrasonography, CT scan and Angiography are widely used, showing over 90% reliability. In this series, AFP was positive in only 50% of patients and recently in Japan it was positive (more thn 200ng/ml) in about 60% of the patients, but sequential measurement of AFP is useful for diagnosis of hepatocellular carcinoma in cirrhotic patients. I am surprised at the high incidence of CEA-positivity in this series, because in only a few patients was CEA positive in Japan. In this series, 48% of the patients had multicentric tumors, but many of them may be multiple intrahepatic metastases because of the tendency of hepatocellular carcinoma to metastasize through portal vein.

The prognosis of hepatocellular carcinoma is poor in general. In the recently collected series in Japan, however, the 5-year cumulative survival rate was about 30% in hepatectomy cases with resection rate of 60% or more and it was over 50% in cases with curative resection.

I have much interest in the cohort analysis in this paper, showing apparent increase of hepatocellular carcinoma in men born in 1911–1930, suggesting that the development of hepatocellular carcinoma was closely related to World War II.

However, the evidence that the endemic environment during World War II may induce hepatocellular carcinoma was not shown. Further analysis is required for co-morbid diseases, such as a history of hepatitis, and blood transfusion, etc.

Ryuji Mizumoto
Department of Surgery
Mie University
Tsu 514, Japan

